# The C allele of the reactive oxygen species modulator 1 (*ROMO1*) polymorphism rs6060566 is a biomarker predicting coronary artery stenosis in Slovenian subjects with type 2 diabetes mellitus

**DOI:** 10.1186/s12920-020-00845-3

**Published:** 2020-12-10

**Authors:** Miha Tibaut, Sara Mankoč Ramuš, Daniel Petrovič

**Affiliations:** 1 Department of Internal Medicine, Rakičan General Hospital, Ul. dr. Vrbnjaka 6, 9000 Murska Sobota, Slovenia; 2 Faculty of Medicine, Institute of Histology and Embryology, University of Ljubljana, Korytkova 2, 1105 Ljubljana, Slovenia; 3 International Centre for Cardiovascular Diseases MC Medicor d.d., Izola, Slovenia

**Keywords:** Reactive oxygen species modulator 1, Coronary artery disease, Diabetes mellitus, Genetic polymorphism, Biomarker

## Abstract

**Background:**

We aimed to examine the role of the rs6060566 polymorphism of the reactive oxygen species modulator 1 (*ROMO1*) gene in the development of myocardial infarction (MI) in Caucasians with type 2 diabetes (T2DM).

**Methods:**

A total of 1072 subjects with T2DM were enrolled in this cross-sectional case–control study: 335 subjects with MI and 737 subjects without clinical signs of coronary artery disease (CAD). The genetic analysis of the rs6060566 polymorphism was performed in all subjects. To assess the degree of coronary artery obstruction, a subpopulation of 128 subjects with T2DM underwent coronary computed tomography angiography. Next, endarterectomy samples were obtained during myocardial revascularization from diffusely diseased coronary arteries in 40 cases, which were analysed for ROMO1 expression according to their genotype.

**Results:**

There were no statistically significant associations between different genotypes or alleles of the rs6060566 polymorphism and MI in subjects with T2DM. The carriers of the C allele of the *ROMO1* rs6060566 had a threefold increased likelihood of having 50–75% coronary artery stenosis (Adjusted OR = 3.27, 95% CI 1.16–9.20). Subjects with two affected coronary arteries had a 3.72 fold higher prevalence of MI (OR = 3.72, 95% CI 1.27–10.84). With CAD in LMCA or LAD, MI prevalence was about 3.5-fold higher (*p* = 0.07 for LMCA and *p* = 0.01 for LAD). Furthermore, the carriers of the rs6060566 C allele showed higher number of positive cells for ROMO1 expression in endarterectomy samples of coronary arteries.

**Conclusions:**

According to our study, the rs6060566 polymorphism of the *ROMO1* gene is not a risk factor for MI in Caucasians with T2DM. However, we found that subjects carrying the C allele were at a 3.27-fold increased risk of developing severe CAD compared with those who had non-obstructive CAD. Moreover, C allele carriers showed a statistically higher number of cells positive for ROMO1 compared with T allele carriers in coronary endarterectomy samples.

## Background

Type 2 diabetes mellitus (T2DM) is a heterogeneous group of metabolic disorders that affects around 8% of the world population and was thought to be responsible for 5 million deaths in 2015 worldwide [1]. In addition, it is one of the major risk factors for coronary artery disease (CAD), and more than 40% of patients with acute coronary syndrome (ACS) have DM [2]. CAD is characterized by atherosclerosis in epicardial coronary arteries and can be asymptomatic, whereas ACS usually presents with a symptom, such as unstable angina, and is frequently associated with myocardial infarction (MI) regardless of the presence of CAD [3]. Because of the proatherosclerotic, proinflammatory, and prothrombotic states associated with diabetes, diabetic patients with ACS are at high risk of subsequent cardiovascular events [4]. The 7-year risk of developing MI in diabetic patients was comparable to the risk of MI in non-diabetic patients with prior MI, which suggests that diabetes contributes significantly to the development of MI and can be considered as possible CAD risk equivalent [5].

In a physiological system, the imbalance between antioxidant defence mechanism and reactive oxygen species (ROS) production leads to oxidative stress and subsequent pathological conditions [6]. CAD occurs as a consequence of accelerated atherosclerosis, mainly driven by oxidative stress [7]. Numerous defence genes are involved in maintaining the balance between oxidant production and their removal by ROS-scavenger enzyme systems.

Reactive oxygen species modulator1 (*ROMO1*) gene is located in chromosome 20q11.22 [8] and produces a small transmembrane protein located in the inner mitochondrial membrane. It is a unique nonselective cation channel that is suggested to be regulated in response to fluctuation in free iron concentration and of its redox state [9]. Nevertheless, ROMO1 is vital for normal mitochondrial morphology and function [10], while its activation induces ROS production in the mitochondrial respiratory chain leading to oxidative stress and cell death [11]. Moreover, ROMO1 is involved in cell proliferation [12], cell apoptosis [13], it is thought to have a role in replicative senescence [14] as well as in carcinogenesis and tumour progression [15].

*ROMO1* gene and its polymorphisms are relatively understudied. The role of ROMO1 in oxidative stress is well established. In addition, current knowledge on pathophysiology of CAD and MI sets oxidative stress as one of the key pathogenic mechanisms in its development [7] which is more prominent in T2DM subjects. Up until now, only one study has examined the relationship between *ROMO1* polymorphism and vascular complications of T2DM [16]. Therefore, our study aimed to assess the potential role of the *ROMO1* polymorphism rs6060566 in the development of MI in Slovenian subjects with T2DM. Furthermore, we also explored ROMO1 expression in coronary endarterectomy specimens with immunohistochemical staining.

## Methods

### Subjects

This retrospective cross‐sectional case–control study enrolled 1072 unrelated Caucasians with T2DM of at least 10 years’ duration. Participants were divided into two study groups: 335 subjects with MI and 737 subjects with no history of CAD, no signs of ischemic changes on electrocardiogram and no ischemic changes during submaximal stress testing; however, in these control subjects CAD could be clinically silent. Subjects were classified as having T2DM according to the current American Diabetes Association criteria [17]. The diagnosis of MI was made according to the established universal criteria [18]. Subjects with MI were included in the study 1–9 months after the acute event. Subjects without T2DM were not enrolled, because they have lower incidence of MI and lower levels of oxidative stress. Consequently, their inclusion would confound the presumed relation of MI to *ROMO1* polymorphism.

Further, to assess the degree of coronary artery obstruction, a subpopulation of 128 subjects from both groups with T2DM underwent coronary computed tomography angiography (CTA) for diagnostic purposes at the International Centre for Cardiovascular Diseases MC Medicor, Izola, Slovenia. Subjects from the original control group (104 subjects) who were included in this substudy had normal echocardiography at rest and, therefore, low clinical likelihood of obstructive CAD according to 2019 European Society of Cardiology (ESC) guidelines for the diagnosis and management of chronic coronary syndromes. Subjects from original MI group (24 subjects) had coronary CTA prior to MI and, therefore, prior to the inclusion in this study. Non-invasive visualization of the epicardial coronary artery tree and the detection of stenosis were performed on dual source Dual energy CT scanner (Siemens, Germany). The acquisition and reading of the coronary CT angiograms were assessed by B.C., a senior expert cardiac radiologist. Normal coronary arteries were defined by the absence of obstructive or non-obstructive atherosclerotic plaque in the epicardial coronary tree. Non-obstructive CAD was defined by the presence of plaque occupying a cross-sectional area stenosis < 50%. The severity of CAD was classified by the degree of stenosis of the cross-sectional area (< 50%, ≥ 50—% ≤ 75% and > 75%) and by the number of diseased vessels (score from 0 to 3; as 0 for no vessel disease (VD), 1 for single VD, 2 for double VD and 3 for triple VD). Angiographically diagnosed diseased left main coronary artery (LMCA) was scored as 1 whilst ignoring stenosis of any of the two major branches: left anterior descending (LAD) or left circumflex (LCx). In addition, if LMCA was not affected by atherosclerosis, we assigned score 1 for each LAD or/and LCx, respectively. At last, diseased right coronary artery (RCA) was scored as 1.

All subjects enrolled in the study were of Caucasian ethnicity. After an informed consent for the participation in the study was obtained, a detailed interview (Additional file 1: The questionnaire) was made including active smoking status. For assessing the cardiovascular risk status of individual subject the Framingham equitation was used (Additional file 1: Cardiovascular risk assessment) and past or current comorbid conditions were taken into account (Additional file 1: Other comorbidities).

Furthermore, their blood was drawn for biochemical analysis and genotyping. Body mass index (BMI) was calculated as weight in kilograms divided by the height in meters square.

### Biochemical analyses

Fasting glucose, high total cholesterol, low density lipoproteins (LDL), high density lipoproteins (HDL), and triglycerides were determined by standard colorimetric assays on an automated biochemistry analyser (Ektachem 250 Analyser, Eastman Kodak Company, Rochester, MN, USA). The serum level of low density lipoprotein (LDL)-cholesterol was calculated by the Friedewald formula (Friedewald et al. 1972). Hyperlipidaemia was defined as total cholesterol higher than 5 mmol/L and/or triglycerides higher than 2 mmol/L, or as a condition treated with hypolipidemic medications.

Glycated haemoglobin (HbA1c) values (non-diabetic reference range of 3.8–5.3%) were estimated by high-performance liquid chromatography. Three recent HbA1c levels were averaged for each subject. High sensitivity C-reactive protein (hsCRP) was measured using a latex enhanced immunonephelometric assay.

Moreover, we assessed oxidative stress in a subgroup of 115 subjects with T2DM with no experience of MI (23 with CAD (CAD+) and 92 without CAD (CAD−) by examining the serum levels of 8-hydroxy-2′-deoxygunosine (8-OHdG), a known marker for oxidative DNA damage. 8-OHdG levels were measured by commercially available enzyme-linked immunosorbent assay (ELISA) kit (IBL International GmbH, Hamburg, Germany) according to the manufacturer’s instructions. The ELISA kit uses a highly sensitive monoclonal antibody against 8-OHdG with an assay sensitivity of 0.5 ng/mL and intra- and interassay coefficients of variation of less than 10%.

### Genotyping

Genomic DNA was extracted from 100 μL of whole blood using a Qiagen isolation kit. The rs6060566 polymorphism of the *ROMO1* gene was genotyped by KBioscience Ltd using their own novel fluorescence‐based competitive allele‐specific PCR (KASPar) assay. Details of the method used can be found at http://www.kbioscience.co.uk/.

### Immunohistochemistry

In the second part of the study we included 40 subjects with T2DM with angina pectoris who had surgical myocardial revascularization. Coronary endarterectomy tissue samples were obtained during myocardial revascularization from diffusely diseased coronary arteries. With respect to different rs6060566 genotypes, relative expression for ROMO1 in resected tissue samples was analysed by immunohistochemistry.

Sections from formalin-fixed, paraffin-embedded tissue blocks of 40 coronary endarterectomy specimens were cut at a thickness of 5 µm. Immunohistochemistry was carried out using a VENTANA BenchMark Ultra Slide Staining System (Roche Diagnostics). Endarterectomy specimens were stained with antibody against ROMO1 (1:100, rabbit polyclonal, HPA012782, Sigma Prestige Antibodies, St. Louis U.S.A.). The detection of primary rabbit immunoglobulins was carried out with the NovoLink Max Polymer Detection System (Leica Biosystems Newcastle Ltd, United Kingdom) following the manufacturer’s instructions. The sections were further incubated with chromogen diamonobenzidine (DAB). Subsequently, reaction with the peroxidase produced a brown precipitate at the ROMO1 sites. Using a light microscope (Axio Scope 2, Zeiss Group, Germany), two researchers P.N. and D.P. independently evaluated the slides and manually counted ROMO1 positive cells at 400 × magnification. Numerical areal density of cells that were immunoreactive for ROMO1 was calculated (the number of positive cells per mm^2^) as described before [19].

### Statistical analysis

Normally distributed continuous variables were expressed as means ± standard deviations, and as median (interquartile range) when asymmetrically distributed. The normality of the continuous variables was examined by the Kolmogorov–Smirnov test. Normally distributed continuous variables were tested using an unpaired Student̕ s *t* test, and the Mann–Whitney U-test when asymmetrically distributed. Discrete variables were compared with Pearson χ^2^ test, which was also used to test whether the genotypes distribution departures form Hardy–Weinberg equilibrium. However, when in contingency table cells with expected frequencies < 5 were identified, the Fisher̕ s Exact test was used to determine if there is a significant relationship between two categorical variables.

Additionally, all variables that showed significant differences by univariate analysis were put into a stepwise multiple logistic regression.

The area-under-curve (AUC) of the receiver operating characteristic (ROC) curve for oxidative stress biomarker 8-OHdG was used to characterize utility for discriminating between CAD+ and CAD– among 115 subjects with T2DM and among 34 rs6060566 C allele carriers.

In order to provide the predictive power of the final multivariate logistic regression model, ROC curve analysis and the likelihood ratio test were applied.

A *p* value of < 0.05 was considered to be statistically significant. Statistical analysis was performed using the SPSS program version 19 (SPSS Inc. Chicago, IL).

## Results

The clinical characteristics and biochemical parameters of the Slovenian subjects with T2DM are listed in Table 1. Cases (335 subjects with MI) had lower BMI, lesser waist circumference and better-controlled hypertension. Additionally, they had a higher total and LDL-cholesterol, triglycerides, and lower HDL-cholesterol. Moreover, cases had longer duration of T2DM. The two groups of subjects were well-matched with regard to age, gender, fasting glucose, HbA1c, hsCRP level and concomitant history of cerebrovascular insult (CVI) or transitory ischemic attack (TIA).

**Table 1:** Demographic and clinical characteristics of cases and controls in Slovenian subjects with T2DM

	Cases (Myocardial infarction)	Controls (Without CAD)	*p* value
Number	335	737	
Age (years)	64.33 ± 9.79	64.12 ± 9.08	0.75
Male gender (%)	200 (59.7)	399 (54.1)	0.09
BMI (kg/m^2^)	29.64 ± 4.14	30.69 ± 4.59	**< 0.001**
Waist circumference (cm)	104.99 ± 11.42	107.86 ± 12.73	**0.02**
Systolic blood pressure (mm Hg)	148.06 ± 19.75	150.78 ± 19.74	0.05
Diastolic blood pressure (mm Hg)	82.11 ± 10.59	84.63 ± 11.52	**< 0.001**
DM duration (years)	15 (10–23)	13 (9–18)	**< 0.001**
Fasting glucose (mmol/L)	8.87 ± 2.90	8.60 ± 2.54	0.25
HbA1c (%)	7.88 ± 1.34	7.50 ± 1.9	0.64
Total cholesterol (mmol/L)	5.15 ± 1.45	4.64 ± 1.12	**< 0.001**
HDL-cholesterol (mmol/L)	1.14 ± 0.30	1.24 ± 0.35	**< 0.001**
LDL-cholesterol (mmol/L)	2.94 (2.22–3.76)	2.50 (2.02–3.10)	**< 0.001**
Triglycerides (mmol/L)	1.90 (1.34–2.70)	1.60 (1.10–2.43)	**< 0.001**
Smoking prevalence (%)	43 (22.8)	66 (9.0)	0.05
CVI (%)	27 (8.1)	44 (6.0)	0.20
TIA (%)	17 (5.1)	21 (2.8)	0.07
hsCRP (mg/L)	2.40 (1.28–4.80)	2.40 (1.30–3.90)	0.15

Values in bold indicate statistical significance

*BMI* body mass index, *HbA1c* glycated haemoglobin, *CVI* cerebrovascular insult, *TIA* transitory ischemic attack, *hsCRP* high-sensitivity C-reactive protein

To address the issue of the oxidative stress status for CAD in subjects with T2DM without history of previous MI, the oxidative damage of DNA was evaluated by measuring the serum level of 8-OHdG. However, in the subgroup of 115 subjects with T2DM no significant difference in the median serum level was observed when 23 subjects with CAD (CAD+) and 92 subjects without CAD (CAD−) were compared [1.47 (range: 1.31–1.71) vs. 1.51 (1.3–1.73) ng/mL], respectively (*p* = 0.80, Mann–Whitney U-test). In addition, among the 34 rs6060566 C allele carriers, median serum level was higher in 9 CAD+ subjects when compared to 25 CAD− subjects [1.54 (range: 1.4–1.72) vs. 1.48 (1.33–1.69) ng/mL], respectively, but the difference was not statistically significant (*p* = 0.48, Mann–Whitney U-test).

Furthermore, the ROC curve analysis was used to evaluate if the serum level of 8-OHdG could serve as a biomarker for CAD. In neither of the examined groups, 8-OHdG had the ability to correctly classify subjects with T2DM as having CAD or not. Specifically, in each of the two groups, 8-OHdG yielded AUC values of 0.483 (95% CI 0.35–0.61) and 0.655 (95% CI 0.37–0.79), respectively, with *p* values far above the significance cutoff value (*p* = 0.05, ROC curve analysis).

Moreover, we found that oxidative stress in 34 C allele carriers (CC + CT genotypes) was not significantly different from the 81 TT carriers [1.49 (range: 1.36–1.71) vs. 1.51 (1.26–1.71) ng/mL], respectively (*p* = 0.70, Mann–Whitney U-test).

The genotype and allele frequencies of the *ROMO1* rs6060566 polymorphism are shown in Table 2. Genotype distributions for both cases (subjects with MI) and controls (subjects without CAD) were in Hardy–Weinberg equilibrium (cases: *p* = 0.70; controls: *p* = 0.83, Pearson χ^2^ test; respectively). Moreover, in each of the studied groups, genotype (cases: *p* = 0.40 and controls: *p* = 0.21, Pearson χ^2^ test) and allele (cases: *p* = 0.24 and controls: *p* = 0.1, Pearson χ^2^ test) frequencies were not significantly different from those reported for the datasets in the 1000 Genomes Project Phase 3 European population.

**Table 2:** Genotype and allele frequencies distribution of the rs6060566

		Cases (%) (N = 335)	Controls (%) (N = 737)	*p* value
Genotypes	CC	10 (3.0%)	21 (2.8%)	0.98
	CT	90 (26.9%)	202 (27.4%)	
	TT	235 (70.1%)	514 (69.7%)	
Alleles	C allele (%)	110 (16.4%)	244 (16.6%)	0.94
	T allele (%)	569 (83.6%)	1230 (83.4%)	
*p* (HWE)		0.70	0.83	

*HWE* Hardy–Weinberg equilibrium, *MAF* minor allele frequency

Moreover, binary logistic regression analyses for different genetic models found no significant associations between different genotypes or alleles of the rs6060566 polymorphism and the risk of MI in Slovenian subjects with T2DM. Estimates of ORs were adjusted (adjusted ORs) (Table 3) for the variables (BMI, waist circumference, diastolic blood pressure, total cholesterol, HDL- and LDL-cholesterols, triglycerides, duration of DM in years) that were significant in the univariate analyses (Table 1).

**Table 3:** Binary logistic regression analyses for the association between rs6060566 of the *ROMO1* and MI in Slovenian subjects with T2DM

Genetic model	Cases/controls	Adjusted OR (95% CI)	*p* value
Co-dominant			
CC versus TT^a^	10/21 versus 235/514	1.12 (0.12–10.83)	0.92
CT versus TT^a^	90/202 versus 235/514	1.68 (0.84–3.36)	0.14
Dominant			
[CC + CT] versus TT^a^	100/223 versus 235/514	1.64 (0.83–3.22)	0.15
Recessive			
CC versus [CT + TT]^a^	10/21 versus 325/716	0.96 (0.109.17)	0.97

^**a**^The reference; OR, odds ratio; adjusted OR for BMI, waist circumference, diastolic blood pressure, total cholesterol, HDL- and LDL-cholesterols, triglycerides, duration of DM in years; CI, confidence interval

Subjects who underwent coronary CTA did not show any significant differences in genotype and allele frequency distribution for the rs6060566 polymorphism (Table 4).

**Table 4:** Genotype and allele frequency distributions of the rs6060566 polymorphism in 128 subjects with T2DM who underwent coronary CTA

	Number of diseased vessels				*p* value	Percentage of cross-sectional area stenosis			*p* value
	None	1 VD	2 VD	3 VD		< 50%	≥ 50% ≤ 75%	> 75%	
	N (%)	N (%)	N (%)	N (%)		N (%)	N (%)	N (%)	
CC	2 (4.2)	0	0	0	0.895*	2 (2.1)	1 (3.6)	0	0.262*
CT	14 (29.2)	4 (18.2)	10 (25)	4 (25)		25 (25.8)	12 (42.9)	1 (33.3)	
TT	33 (66.7)	18 (81.8)	31 (75)	12 (75)		70 (72.2)	15 (53.6)	2 (66.7)	
C	18 (18.4)	4 (9.1)	10 (12.2)	4 (12.5)	0.44†	29 (14.9)	14 (15)	1 (16.7)	0.213†
T	80 (81.6)	40 (90.9)	72 (87.8)	28 (87.5)		165 (85.1)	42 (75)	5 (83.3)	

*VD* vessel disease

*The *p* value was obtained with Fisher̕ s Exact test

^†^The *p* value was obtained with Pearson χ^2^ test

We performed multinomial logistic regression analyses to evaluate the association of the rs6060566 polymorphism with CAD in these subjects. Because of the low frequency of the minor C allele (Table 4) the analyses were performed assuming the dominant genetic model ([CC + CT] vs. TT). The final model is shown in Table 5. The dependent variables describing the severity of CAD were the number of diseased vessels and extent of stenosis (no diseased vessel and stenosis < 50% were used as references, respectively). Independent variables included in the model were dominant genetic model (TT genotype was used as reference), age, gender, lipid parameters and duration of T2DM in years. We did not observe any interactions between the dominant genetic model and CAD without adjustment for the possible confounders (Table 5). Nevertheless, when well-known CAD risk factors (age, gender, lipid parameters and duration of T2DM in years) were fixed in the model, the association between carriers of the [CC + CT] genotypes and ≥ 50% ≤ 75% cross-sectional area stenosis became statistically significant (*p* = 0.025, multinomial logistic regression). The carriers of the C allele of the *ROMO1* rs6060566 had a threefold increased likelihood of having coronary artery stenosis (Adjusted OR = 3.27, 95% CI 1.16–9.20, Table 5). However, in a full fitted model, only dominant genetic model with CC + CT genotypes (Wald = 5.05, *df* = 1, *p* = 0.025, multinomial logistic regression) and triglycerides (Wald = 3.77, *df* = 1, *p* = 0.05, multinomial logistic regression) were independently associated with CAD in subjects who were diagnosed with ≥ 50%–≤ 75% stenosis. The model demonstrated the independent strength of the effect of the C allele; thus it appears that rs6060566 of the *ROMO1* gene may contribute to CAD risk. Furthermore, the fit of the multivariate model was tested by ROC curve analysis. The predictive power of the final model, which included a dominant genetic model and additional risk factors—age, gender, lipid parameters and duration of T2DM—with the AUC of 0.729 (95% CI 0.61–0.85), was statistically significant (*p* < 0.001, ROC curve analysis). In addition, likelihood ratio test as a measure of the statistical significance of all predictors in the model showed a statistically significant score with the χ^2^ value of 27.62 (*df* = 16, *p* = 0.035, likelihood ratio test).

**Table 5:** Multinomial logistic regression analyses for the association between dominant genetic model ([CC + CT] vs. TT^a^) of the rs6060566 polymorphism and CAD in 128 subjects with T2DM who underwent coronary CTA

	OR (95% CI)	*p *value	Adjusted OR (95% CI)	*p *value
≥ 50% ≤ 75%	2.25 (0.95–5.34)	0.067	3.27 (1.16–9.20)	**0.025**
> 75%	1.3 (0.11–14.89)	0.835	0.94 (0.03–25.8)	0.969
1 VD	0.44 (0.08–2.56)	0.364	0.25 (0.02–2.58)	0.245
2 VD	0.67 (0.18–2.5)	0.547	0.88 (0.12–6.39)	0.899
3 VD	0.66 (0.11–4.08)	0.661	0.85 (0.03–2.78)	0.928

OR, odds ratio. ORs were adjusted for age, gender, lipid parameters and duration of T2DM in years. The statistically significant result (*p* value < 0.05) is highlighted in bold

^a^The reference

**Fig. 1:**
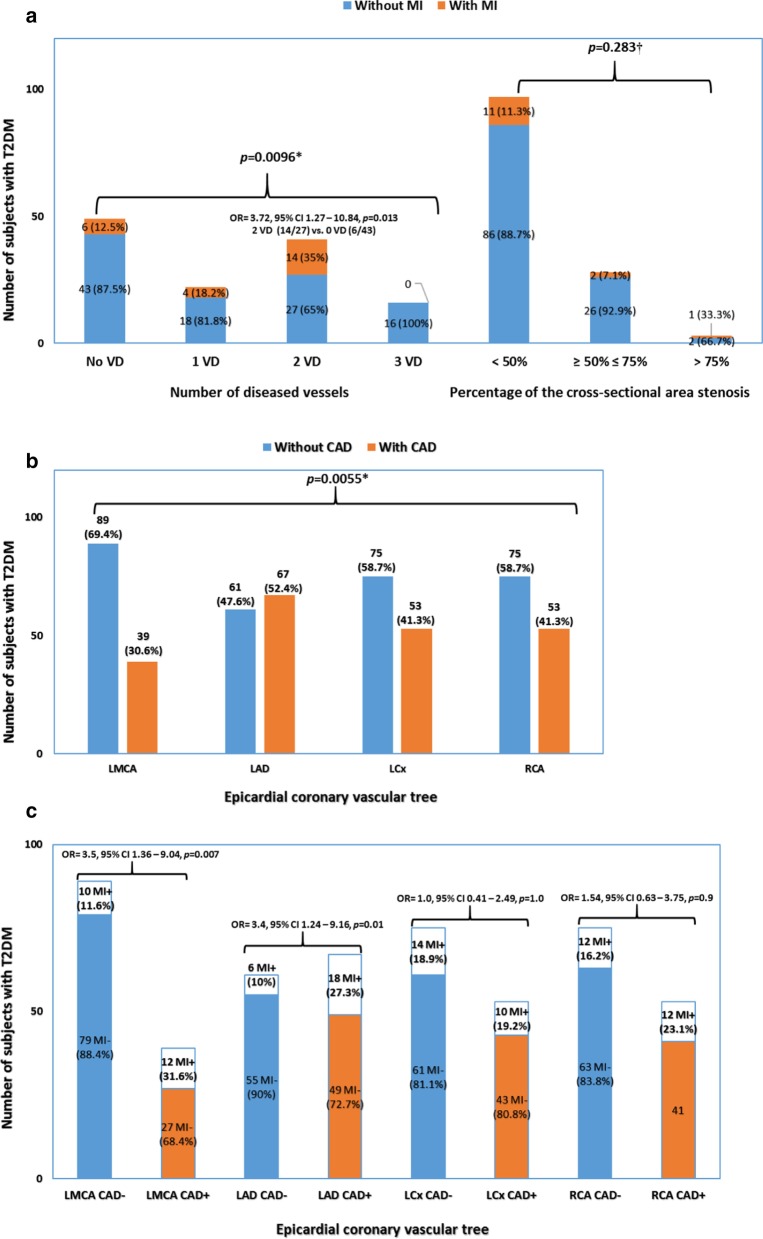
Findings at coronary CTA. Distribution of Slovenian subjects with T2DM according to the extent and severity of CAD (**a**). Atherosclerotic burden in major coronary arteries (**b**). Relation between specific coronary artery and the risk of MI in subjects with CAD (CAD+) in comparison with subjects without CAD (CAD−) (**c**)

A total of 128 subjects with T2DM underwent coronary CTA (Fig. 1a–c). A single VD (1 VD) was observed in 22 (17%) subjects, two VD (2 VD) in 41 (32%) and three VD (3 VD) in 16 (13%) subjects. Moreover, in 49 (38%) subjects all major epicardial coronary arteries (LMCA, LAD, LCx and RCA) on CT angiograms (Fig. 1a) were normal. Moreover, 97 (76%) subjects had non-obstructive CAD (cross-sectional area stenosis of < 50%), in 28 (22%) subjects a cross-sectional area stenosis of ≥ 50%–≤ 75% was detected while only 3 subjects (2%) had stenosis of > 75% (Fig. 1a). As shown in Fig. 1a, subjects with 2 VD (14/41, 35%) and non-obstructive CAD (11/95, 11.3%) suffered from nonfatal MI more often than other subjects in both comparative groups (number of diseased vessels and percentage of cross-sectional area stenosis). Of note, there was a statistically significant difference (*p* = 0.0096, Pearson χ^2^ test) in the frequency distribution between subgroups with and without MI with regard to the extent of the CAD (Fig. 1a). In contrast, no difference (*p* = 0.283; Fisher’s Exact test) was observed between subgroups with regard to the coronary cross-sectional area stenosis (Fig. 1a). With regards to the number of the involved vessels, a significantly higher frequency (*p* = 0.013; Pearson χ^2^ test) of MI was found in subjects with 2 VD. Interestingly, subjects with two affected epicardial coronary arteries showed a 3.72-fold risk for MI (OR = 3.72, 95% CI 1.27–10.84, Fig. 1a).

Coronary CTA revealed that more than 50% of the 128 subjects had developed CAD in LAD (Fig. 1b), while the remainder of the epicardial coronary arteries were spared of atherosclerotic disease more frequently. Atherosclerotic changes were noticed in LMCA in 39 subjects (30.6%), while a slightly higher percentage of atherosclerotic disease was seen in LCx and RCA (41%) (Fig. 1b). Furthermore, the relationship between the presence or absence of CAD with regards to coronary arteries was statistically significant (*p* = 0.055, Pearson χ^2^ test; Fig. 1b).

As depicted in Fig. 1c, subjects with CAD (CAD+) in LMCA or LAD had about 3.5-fold higher risk of experiencing MI (*p* = 0.07 for LMCA and *p* = 0.01 for LAD, Pearson χ^2^ test; respectively) compared with subjects without CAD (CAD−). However, in subjects with diseased LCx and RCA, MI occurred more frequently than in subjects with disease-free epicardial coronary arteries, although the difference was not statistically significant (*p* = 1.0 for LCx and *p* = 0.9 for RCA, Pearson χ^2^ test; respectively).

**Fig. 2:**
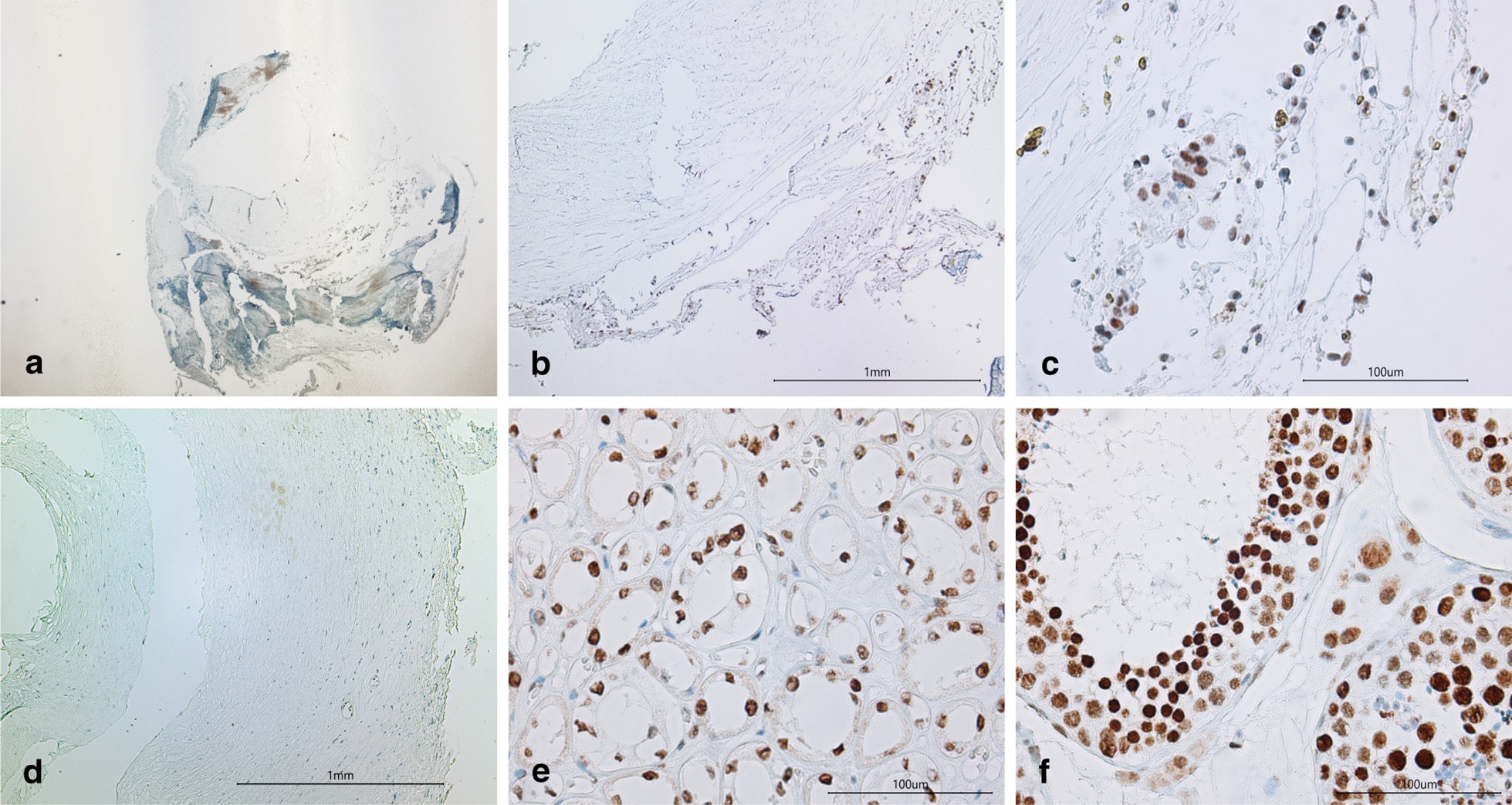
Histology and immunohistochemistry for ROMO1 in endarterectomised tissue samples of coronary arteries (**a**–**d**) and positive controls (**e–f**): ROMO1 positive reaction in human kidney medulla (**e**) and testis (**f**). ROMO1 expression in representative tissue section in the C allele carrier of the rs6060566 polymorphism (**a**–**c**), **c** is a higher magnification of the square in **b**. A portion of the coronary artery (**c**) in which immunohistochemistry for ROMO1 reveals positive brown cells (arrows). No staining of cells in the coronary artery of the T allele carrier (**d**). Original magnification × 25 (**a**), × 100 (**b**, **d**, **e**) and × 400 (**c**, **f**)

At the end of this study, the coronary artery segments, which were obtained by endarterectomy from subjects with advanced atherosclerosis, were examined with immunohistochemical staining. A statistically significantly higher numerical areal density of ROMO1 positive cells was found in 17 subjects with the C allele (Fig. 2) in comparison with 23 subjects with ROMO1 TT genotype (wild type) (835 ± 215/mm^2^ versus 412 ± 153/mm^2^; *p* < 0.001, Student̕ s *t* test).

## Discussion

Our study investigated the role of oxidative stress *ROMO1* gene polymorphism rs6060566 in Slovenian subjects with T2DM who experienced MI. We found no association of the rs6060566 polymorphism with MI. In contrast, we have identified a significant relationship between carriers of the [CC + CT] genotypes (under the assumption of the dominant genetic model) and ≥ 50%–≤ 75% cross-sectional area stenosis. Thus, we found that subjects carrying the C allele were at a 3.27-fold increased risk of developing severe CAD compared with those who had non-obstructive CAD. This contradiction in our results may be attributed to a slightly different pathophysiology of CAD and MI [7, 20]. CAD is a slowly progressive disease with inflammation and oxidative stress as main pathophysiologic factors, while MI is a complication of stable CAD caused by superficial plaque erosion, plaque rupture, erosion from calcium nodules or intraplaque haemorrhage. These triggers can be explained only in part by oxidative stress [20], and depend more on plaque vulnerability. Moreover, only 14% of occlusions leading to MI develop at sites of pre-existing high-grade stenosis (more than 70% in diameter) and more than two thirds of MIs occur on non-obstructive lesions (< 50% in diameter) [20].

To continue, a significantly higher frequency of MI was found in subjects with 2 VD as compared to the subjects in whom CAD was not detected by CTA. At first glance, it seems that the frequency of subjects with MI increased with the number of involved vessels. Our results are partly supported by in-depth study conducted on 12.594 diabetes patients by Gyldenkerne et al. [21] who found that the extent of CAD is a major risk factor for MI and death in patients with DM. We could not confirm this increased risk for MI in a group of subjects with 3 VD, most likely because of the small sample size. Another contributor could be our definition of the number of diseased vessels when LMCA was affected, which gave score of 1 disregarding LAD or LCX involvement. Although it is known that diabetic patients suffer from more extensive CAD and hence higher incidence of multi-vessel CAD than non-diabetic subjects [22], in our research, 3 VD was diagnosed only in 16 out of 128 subjects who underwent coronary CTA. It is important to note that all of them were diagnosed without atherosclerotic lesions in LMCA (due to the above-mentioned definition) and none of them has suffered a MI. Furthermore, all 16 subjects underwent either percutaneous coronary intervention (PCI) or coronary artery bypass grafting surgery (CABG). We may assume that appropriate myocardial revascularization strategy with optimal medical therapy led to reduced incidence of MI in this small group of Slovenian subjects with 3 VD. Recently, a large-scale meta-analysis based on 18.224 patients with DM undergoing PCI and CABG has demonstrated the superiority of CABG in reducing mortality, MI and the need for repeat revascularization in patients with DM and complex CAD (including LMCA and/or multivessel disease) [23]. However, our study did not examine clinical outcomes of PCI or CABG in subjects with complex CAD.

In our study, subjects with CAD localized in LMCA and LAD had a significantly higher occurrence of nonfatal MI compared to subjects without CAD in respective epicardial coronary arteries. In our study, they had about a 3.5-fold higher risk of MI. Our observations parallel with well-known facts that diabetes approximately doubles the risk of MI and death among patients with known CAD [24, 25]. On the other hand, the current study shows that MI is less frequent in subjects with disease-free epicardial coronary arteries. Thus, in the absence of angiographically significant CAD, patients with diabetes treated with contemporary prophylactic therapy have the same risk of cardiovascular events as patients without diabetes [26]. Therefore, optimal medical therapy and appropriate selection of myocardial revascularization strategy is critical for patients with DM [27].

T2DM patients are strongly prone to atherothrombotic complications in epicardial coronary arteries as well as in other macro- and microvasculature. One of the major contributors to susceptibility for atherothrombotic complications is genetic background [28]. Many studies [29, 30] have shown an association of different genetic loci and their polymorphisms with higher risk of atherosclerosis, CAD and MI. Numerous identified risk polymorphisms have no known pathophysiological function, but others are involved in inflammatory response, lipid function, transportation, endothelial dysfunction or oxidative stress regulation.

As oxidative stress is recognized as a central pathogenic process in accelerated atherosclerosis in T2DM, various studies [7] investigated the associations of oxidative stress genes (e.g. NADPH1 oxidase, Myeloperoxidase, Glutathione peroxidase 1, Glutathione S-transferase [31], NAD(P)H1: Quinone oxidoreductase, Superoxide dismutase 1 and 2 [32], Thioredoxin reductase 2 [33], Uncoupling protein 2 [34], etc.) with micro- and macrovascular complications in T2DM. In general, studies yielded conflicting results; some of these discrepancies could be attributed to different study populations, races, small sample sizes, study types (i.e. retrospective) and design. It should be noted that many association studies were conducted on one single common polymorphism and hence have not scanned for the gene–gene or gene-environment influences on the risk for MI.

Oxidative stress occurs when ROS production exceeds the elimination capacity of the antioxidant system. Most ROS are generated in the mitochondrial respiratory chain [35] and although by-products, they are vital for normal processes, such as the maintenance of the vascular tone, cell adhesion, immune responses and cellular growth [36]. Increased expression of ROMO1, firstly identified in tumour cells, increases ROS production [35], leading to oxidative stress and cell death [11]. The product encoded by *ROMO1* is a small transmembrane protein located in the inner mitochondrial membrane that has been recently found to be a unique nonselective cation channel. Its function is suggested to be regulated in response to fluctuation of free iron concentration and redox state of iron [9]. It is proposed that ROMO1 activation causes the recruitment of B-cell lymphoma-extra-large (Bcl-xL) protein to the outer mitochondrial membrane that in turn reduces its membrane potential, resulting in ROS production [13].

ROMO1 is an essential protein involved in several cell functions. It is vital for maintaining mitochondrial cristae shape [10, 37] and its loss causes mitochondrial cytochrome c leakage, which is one of the key molecules involved in the intrinsic apoptotic pathway [37]. Its role in tumour necrosis factor (TNF)-alpha-induced apoptosis was furthermore confirmed by Kim et al. [13].

Hwang et al. [11] showed that enforced ROMO1 expression leads to massive cell death (necrosis) by excessive ROS production. It was shown that in physiological states ROMO1-derived ROS were indispensable for the proliferation of both normal and cancer cells, respectively [12]. Moreover, Chung el al [37] showed that ROMO1 might be a principal factor in the deregulation of nuclear factor-κB and related pathways that contribute to tumour cell proliferation and invasion. It is also thought to have a role in replicative senescence [14]. Lung, colorectal cancer and gliomas [38] have been linked to ROMO1. In non-small cell lung cancer ROMO1 can serve as disease biomarker and is a predictor of poor survival and malignant effusions in these patients [39, 40]. In colorectal cancer, ROMO1 expression predicts poor survival and higher invasiveness [15]. Its role is also being investigated concerning pulmonary and renal fibrosis [41], obstructive sleep apnoea syndrome [42] and Fanconi anaemia [43].

Furthermore, to our knowledge, this is the first study investigating the expression of the ROMO1 in coronary endarterectomy samples. The C allele carriers showed statistically higher number of cells positive for ROMO1 compared with T allele carriers. This result corroborates the findings by Petrovič et al. [16], in which greater ROMO1 expression was found in fibrovascular membranes of subjects with microvascular complication of T2DM, namely proliferative diabetic retinopathy. *ROMO1* rs6060566 polymorphism is located in an intron region. Therefore, the exact mechanism on how rs6060566 polymorphism enhances the expression of this gene is not known. Several possible processes can be affected: transcription rate, nuclear export, transcript stability or, moreover, introns can also increase the efficiency of mRNA translation [44].

To continue, we assumed that *ROMO1* rs6060566 polymorphism might be associated with MI. However, we failed to show the presumable association in Slovenian subjects with T2DM. One possible explanation for our results might be that some of the controls had clinically silent CAD that we did not detect with our inclusion criteria, or had other macro- or microvascular complications that could also be induced by oxidative stress. Not detecting clinically silent CAD is therefore a major limitation of our study. Otherwise, subjects with MI had a longer duration of DM and higher levels of HbA1c. It is generally believed that the relative risk for MI increases with any increase in glycaemia above the normal range [45]. On the other hand, blood pressure in cases was better managed, which is probably a consequence of a recent MI, thus recently optimized medical therapy and presumed better compliance.

## Conclusions

In conclusion, in this study we did not observe any association between the rs6060566 polymorphism of the *ROMO1* gene and the risk of MI in Caucasians with T2DM. However, we found that subjects carrying the C allele were at a 3.27-fold increased risk of developing severe CAD compared with those who had non-obstructive CAD. Moreover, C allele carriers showed a statistically higher number of cells positive for ROMO1 compared with T allele carriers in coronary endarterectomy samples. Furthermore, it seems likely that the extent of CAD was a risk factor for MI in a subgroup of subjects in whom 2 VD was diagnosed with coronary CTA.

In summary, our findings signal that 8-OHdG, a well-known oxidative stress marker, does not have the ability to discriminate between subjects with and without CAD. Association analyses in different ethnic populations are needed to confirm and validate our preliminary findings.

New opportunities for research activity are needed to explore the contribution of oxidative stress to the perpetuation of the atherosclerotic process in the coronary artery system in subjects with T2DM.

## Supplementary Information

**Additional file 1.** The questionnaire, cardiovascular risk assessment Table S1 and information on other comorbidities Table S2.

**Additional file 2.** Demographic, clinical and genetic data of Slovenian subjects with T2DM.

## Data Availability

The demographic, clinical, and genetic data that support the findings of this study are available under Additional file 2. The SNP data is available in European Variation Archive on https://www.ebi.ac.uk/eva/?eva-study=PRJEB41691.

## Abbreviations

ACS: Acute coronary syndrome
OR: Odds ratio
AUC: Area-under-curve
Bcl-xL: B-cell lymphoma-extra-large
BMI: Body mass index
CABG: Coronary artery bypass grafting surgery
CAD: Coronary artery disease
CI: Confidence interval
CTA: Computed tomography angiography
CVI: Cerebrovascular insult
DAB: Diamonobenzidine
DM: Diabetes mellitus
8-OHdG: 8-Hydroxy-2′-deoxygunosine
ELISA: Enzyme-linked immunosorbent assay
ESC: European society of cardiology
HbA1c: Glycated haemoglobin
HDL: High density lipoprotein
hsCRP: High sensitivity C-reactive protein
HWE: Hardy–Weinberg equilibrium
KASP: Competitive allele‐specific PCR
LAD: Left anterior descending
LCx: Left circumflex
LMCA: Left main coronary artery
LDL: Low density lipoprotein
MAF: Minor allele frequency
MI: Myocardial infarction
NADP: Nicotinamide adenine dinucleotide phosphate
OR: Odds ratio
PCI: Percutaneous coronary intervention
RCA: Right coronary artery
ROC: Receiver operating characteristic
ROMO1: Reactive oxygen species modulator 1
ROS: Reactive oxygen species
TIA: Transitory ischemic attack
T2DM: Type 2 diabetes mellitus
TNF: Tumour necrosis factor
VD: Vessel disease
