# Supplementary material for: The C allele of the reactive oxygen species modulator 1 (ROMO1) polymorphism rs6060566 is a biomarker predicting coronary artery stenosis in Slovenian subjects with type 2 diabetes mellitus
Source: BMC Med Genomics. 2020 Dec 10;13:184. doi: 10.1186/s12920-020-00845-3 (PMC7731747; doi:10.1186/s12920-020-00845-3)
Supplement: Supplementary file 1 — Additional file 1. The questionnaire, cardiovascular risk assessment Table S1 and information on other comorbidities Table S2. [file 12920_2020_845_MOESM1_ESM.doc]

**The questionnaire**

CORONARY DISEASE/ ATHEROSCLEROSIS/ DIABETES

DATE ___________________________________________________

SURNAME, FIRST NAME_________________________________________AGE_________________

ADDRESS ____________________________________________________________________________

PHONE NR. _________________________________________________ FILE NR. ______________

RESEARCHER NR. _____________________________

CLINICAL DATA:

**CLINICAL DATA FROM DISCHARGE OR OTHER AVALIABLE RECORDS:**

**CORONARY DISEASE (MI, AP, CABG, PCI; WHEN)** NO YES_-WHEN (AGE=

BEFORE 55 YEARS OF AGE AFTER 55 YEARS OF AGE

**CVI** YES NO **TIA** YES NO

**FAMILY HISTORY** **(CD)-**AP/AMI/PCI YES NO BEFORE 55 YEARS OF AGE AFTER 55 YEARS OF AGE

**PAST MEDICAL HISTORY**:

1 HEART FAILURE YES NO

2 FUNCTIONAL NYHA CLASS I II III

3 ALCOHOLISM YES NO

4 SMOKING YES NO

**OBLIGATORY LAB WORK (blood drawn or mailed by regular post)**

Total cholesterol ________________________________________________________

cholesterol LDL ________________________________________________________

cholesterol HDL ________________________________________________________

triglycerides ________________________________________________________

high sensitive CRP __________________________________________________

biochemistry ____ creatinine urea ……

**ADDITIONAL LAB WORK**

Inflammatory markers (fibrinogen, Il-6, Il-8) _____________________________

**THERAPY**

| **Drug** **type** | Drug already prescribed | Commercial drug name | Total daily dose | SIDE EFFECTS |
| --- | --- | --- | --- | --- |
| **statin** |  no  yes |  |  |  |
| fibrate |  no  yes |  |  |  |
| cholesterol absorption inhibitors |  no  yes |  |  |  |
| **ACE inhibitor** |  no  yes |  |  |  |
| ARB (sartan) |  no  yes |  |  |  |
| Direct renin inhibitor |  no  yes |  |  |  |
| **Beta blocker** |  no  yes |  |  |  |
| Ivabradine |  no  yes |  |  |  |
| **Alfa blocker** |  no  yes |  |  |  |
| **Calcium channel blocker** |  no  yes |  |  |  |
| **Loop diuretic** |  no  yes |  |  |  |
| Thiazides |  no  yes |  |  |  |
| Aldosterone antagonists |  no  yes |  |  |  |
| **Indapamide** |  no  yes |  |  |  |
| digitalis |  no  yes |  |  |  |
| nitrates |  no  yes |  |  |  |
| Trimetazidine |  no  yes |  |  |  |
| Propafenone |  no  yes |  |  |  |
| Sotalol |  no  yes |  |  |  |
| Amiodarone |  no  yes |  |  |  |
| Dronedarone |  no  yes |  |  |  |
| Verapamil |  no  yes |  |  |  |
| Diltiazem |  no  yes |  |  |  |
| **Aspirin** |  no  yes |  |  |  |
| Ticlopidine |  no  yes |  |  |  |
| Clopidogrel |  no  yes |  |  |  |
| Prasugrel |  no  yes |  |  |  |
| Dipyridamole |  no  yes |  |  |  |
| Warfarin |  no  yes |  |  |  |
| **peroral antidiabetic** |  no  yes |  |  |  |
| **Insulin** |  no  yes |  |  |  |

**DOPPLER ULTRASOUND OF CAROTID ARTERIES**

**LEFT SIDE**: **DIAMETER ACC DIAMETER ACI DIAMETER ACE_______**

INTIMA MEDIA THICKNESS PROXIMAL FROM ACC BIFURCATION (AT LEAST 3 MEASUREMENTS AND AVERAGE)

INTIMA MEDIA THICKNESS IN ACC BIFURCATION (AT LEAST 3 MEASUREMENTS AND AVERAGE)

**PLAQUES YES / NO**

**SPEED** (PSV/EDV; cm/s): ACC___________ACI___________ACE___________

**PLAQUE DESCRIPTION – ACC, ACC BIFURCATION, ACI, ACE** (thickness, length, area, type 1-5, percent stenosis in regard to hemodynamic criteria)

ACC______________________________________________________________________

ACC BIFURCATION__________________________________________________________

ACI_______________________________________________________________________

ACE_______________________________________________________________________

**RIGHT SIDE**: **DIAMETER ACC DIAMETER ACI DIAMETER ACE_______**

INTIMA MEDIA THICKNESS PROXIMAL FROM ACC BIFURCATION (AT LEAST 3 MEASUREMENTS AND AVERAGE)

INTIMA MEDIA THICKNESS IN ACC BIFURCATION (AT LEAST 3 MEASUREMENTS AND AVERAGE)

**PLAQUES YES / NO**

**SPEED** (PSV/EDV; cm/s): ACC___________ACI___________ACE___________

**PLAQUE DESCRIPTION – ACC, ACC BIFURCATION, ACI, ACE** (thickness, length, area, type 1-5, percent stenosis in regard to hemodynamic criteria)

ACC______________________________________________________________________

ACC BIFURCATION__________________________________________________________

ACI_______________________________________________________________________

ACE_______________________________________________________________________

**FAMILY HISTORY**

Heart diseases, cerebral vascular diseases (CVI, TIA) or peripheral artery disease in relatives (for 3 generations)

| Family relation | Disease | Age at diagnosis | Age at death |
| --- | --- | --- | --- |
|  |  |  |  |
|  |  |  |  |
|  |  |  |  |
|  |  |  |  |
|  |  |  |  |

CORONARY FAMILY HISTORY YES NO BEFORE 55 YEARS OF AGE AFTER 55 YEARS OF AGE

1 HEART FAILURE YES NO

2 FUNCTIONAL NYHA CLASS I II III

3 ALCOHOLISM YES NO

**Table S1. CARDIOVASCULAR RISK ASSESMENT**

| Body height |  |  |
| --- | --- | --- |
| Body weight |  |  |
| Smoking | yes / no |  |
| physical activity (at least 30min, 3-4x weekly) | yes / no |  |
| Type 2 diabetes mellitus | yes / no |  |
| Alcohol (>1 unit for women,>2 units for men) |  | |
| High blood pressure (values) |  | |
| High cholesterol (values) |  | |
| family hypercholesterolemia | yes / no | |
| obesity (BMI** > 25) |  | |
| waist circumference *** (in cm) |  | |
| Framingham Risk Score; 10-year cardiovascular risk assessment |  | |

** BMI body mass index

*** male > 102 cm, female > 88 cm

**Table S2. OTHER COMORBIDITIES**

|  |  | family member with disease | age at diagnosis |
| --- | --- | --- | --- |
| Migraine | yes / no |  |  |
| Depression | yes / no |  |  |
| Rheumatoid arthritis | yes / no |  |  |
| Cancerous diseases |  |  |  |
| Neurological diseases (Alzheimer’s disease, Parkinson’s disease, multiple sclerosis …) |  |  |  |
| Gastrointestinal diseases |  |  |  |
| Kidney disease |  |  |  |
| Pulmonary disease |  |  |  |
| Other psychological disorders |  |  |  |
| Other: |  |  |  |
